# Comparison of Five Different Selective Agar for the Detection of Vancomycin-Resistant *Enterococcus faecium*

**DOI:** 10.3390/antibiotics12040666

**Published:** 2023-03-29

**Authors:** Alessa L. Boschert, Franca Arndt, Axel Hamprecht, Martina Wolke, Sarah V. Walker

**Affiliations:** 1 Institute for Medical Microbiology, Immunology and Hygiene, University Hospital of Cologne, 50935 Cologne, Germany; 2 German Aerospace Center (DLR), Institute of Aerospace Medicine, 51147 Cologne, Germany; 3 German Centre for Infection Research (DZIF), Partner Site Bonn-Cologne, 50441 Cologne, Germany; 4 Institute for Medical Microbiology and Virology, University of Oldenburg, 26001 Oldenburg, Germany; 5 Institute for Clinical Microbiology and Hospital Hygiene, Klinikum Ludwigsburg, 71634 Ludwigsburg, Germany

**Keywords:** vancomycin, *van* gene, enterococcus faecium, screening agar, selective media

## Abstract

Five commercially available selective agar were evaluated regarding sensitivity and specificity to detect vancomycin-resistant *Enterococcus* (*E.*) *faecium*. Altogether 187 *E. faecium* strains were included, comprising 119 *van*-carrying strains (phenotypically vancomycin-resistant *n* = 105; phenotypically vancomycin-susceptible VVE-B *n* = 14) and 68 vancomycin-susceptible isolates. Limit of detection was calculated for each selective agar for pure cultures, stool suspensions and artificial rectal swabs. After 24-h incubation sensitivity ranged between 91.6% and 95.0%. It increased in 2 out of 5 agar after 48-h incubation. Specificity ranged between 94.1% and 100% and was highest after 24 h in 4 out of the 5 agar. Sensitivity of *van*-carrying phenotypically vancomycin-resistant strains was higher after 24 h (97.1–100%) and 48 h (99.1–100%) when compared to *van*-carrying strains that tested vancomycin-susceptible (50.0–57.1% after both incubation periods). Overall, chromID VRE, CHROMagar VRE and Brilliance VRE demonstrated the highest detection rates after 24 h. Detection rates of Chromatic VRE and VRESelect improved after 48 h. Adjustment of incubation time depending on the applied media may be advised. As detection of VVE-B was impeded with all selective agar, screening for vancomycin-resistant enterococci relying solely on selective media would not be recommended for critical clinical samples, but rather in combination with molecular methods to improve detection of these strains. Furthermore, stool samples were demonstrated to be superior to rectal swabs and should be favoured, if possible, in screening strategies.

## 1. Introduction

More than a decade ago, *Enterococcus* (*E.*) *faecium* was defined as part of the so-called ESKAPE group—a group of bacterial species most prone to developing antibiotic resistance [1]. Since then, there have been substantial changes in the prevalence of vancomycin-resistant *E. faecium* (VREfm).

Epidemiological dynamics differ substantially between countries within Europe. In Germany, strains initially detected in 1990 harboured a *vanA*-gene transferring resistance against Vancomycin and Teicoplanin. However, the *vanB* gene conferring resistance against only Vancomycin became the more prevalent resistance gene throughout Germany within the next 20 years. Furthermore, prevalence of VREfm has continued to rise over the past years [2,3]. Since a peak in VREfm prevalence, numbers continually decreased in Portugal until 2019. Since then, they have stagnated on a constant low level of around 8% of all *E. faecium* strains being VREfm [4]. Despite these varying developments, VREfm have been recognised as a serious health problem by several health institutions [5,6].

Although VREfm can also be found in reservoirs outside the hospital environment, previous antibiotic therapy, as well as regular contact with the healthcare system—more precisely previous antibiotic therapy and invasive devices—are associated with a higher colonization rate with VREfm [7]. Since colonization results in an increased infections risk, especially in high-risk patients such as paediatric, haematological patients and those with malignancies [8,9]. Despite several treatment options, such as daptomycin or linezolid, VREfm infections are associated with a higher mortality when compared to infections by vancomycin-susceptible strains [10]. In Europe, deaths ascribed to VREfm have almost doubled from 2007 to 2015 [11]. Similarly, in the USA, vancomycin-resistant enterococci have been estimated to be responsible for 54,500 infections and 5400 deaths in 2017 [12].

Early identification of VREfm in clinical or screening specimen allows for a fast, targeted therapeutic approach and renders a higher cost-effectiveness for healthcare facilities [13,14]—thus, ultimately, further improving patient care. This emphasizes the importance of rapid VREfm detections in diagnostic laboratories as a vital component, not only for infection control [15], but also to guarantee adequate antibiotic therapy strategies. This has also been probated by several national institutions [16,17].

Since the *vanA* phenotype remains the most prevalent, not only in Europe [18], but also worldwide [19], detection of *vanB*-gene VREfm is faced with even higher demands. Since the positive predictive value (PPV) is generally impeded for low prevalence, it is important to assess for screening parameters, including but limited to the PPV. This allows for evaluation of the screening procedure and the potential of false negative results to be integrated in the antimicrobial therapy approach in low as well as high prevalence regions. Furthermore, even though *vanB* VREfm are currently the most prevalent in only some regions, such as Australia and Germany [19], potential future epidemiological shifts may occur—as it has been the case in Germany in the last two decades. These two aspects highlight the necessity to identify potential diagnostic gaps in detection of *vanB*-mediated vancomycin-resistance in enterococci.

Rapid VREfm detection from rectal swabs or other screening specimen may be achieved if these are plated directly on selective media if sensitivity of the employed selective media is sufficient. Yet, screening may be unreliable due to vancomycin-variable strains (VVE). First described in 2011 [20], these strains harbour the *vanA*-gene, yet initially express a vancomycin-susceptible phenotype [21,22]. While they have the ability to switch to a vancomycin-resistant phenotype, they may not only evade automated testing systems [23] but potentially also selective media [24]. While most of the studies describe this phenotypical vancomycin-susceptibility in relation to the *vanA*-gene, similar findings were also made for *vanB* carrying *E. faecium* [25,26], with the phenotypic expression of the resistance being inducible [27]—referred to as VVE-B in the following.

Expression of glycopeptide resistance is controlled by a two-component regulatory system with the membrane-associated domains of the kinase VanS_B_/VanS detecting glycopeptides in the bacterial surrounding. The triggered signal cascade including the response regulator VanR_B_/VanR ultimately results in the synthesis of peptidoglycan precursors with D-Ala-D-Lac termini. Due to this altered site, glycopeptide binding affinity is decreased [28]. This results in a heterogenous expression of vancomycin resistance which not only hampers laboratory screening methods but also targeted antimicrobial therapy. Furthermore, even though each region has a specific predominant *vanB* strain due to heterogeneity within the *vanB* gene cluster, epidemiological shifts have been observed over the past years [29,30]. Vancomycin-resistance itself cannot be inferred from the predominant cluster at a given time due to varying levels of resistance within the clusters themselves [30]. This stresses the need for constant evaluation of laboratory screening methods used in current clinical diagnostics.

Previous studies have either compared several selective media by using clinical samples [31,32,33,34] or evaluated performance of one specific media [25,35,36,37,38]. Yet, detection rate of VVE-B might be difficult to assess by clinical samples, since—due to their heterogenous expression of vancomycin-resistance—they might evade several selective media. Additionally, availability of specific VREfm screening media used in routine diagnostics varies over time.

Therefore, the aim of the study was to compare sensitivity and specificity of VREfm screening agar currently available for VREfm strains but also for strains harbouring a *vanB*-gene while being vancomycin-susceptible (VVE-B), specifically.

## 2. Results

A total of 187 *E. faecium* isolates comprising 68 *van*-gene negative isolates (VVE), 14 vanB-gene carrying phenotypically vancomycin-susceptible isolates (VVE-B) and 105 vancomycin-resistant isolates (VREfm; *vanB*: 84, *vanA*: 20, *vanA*/*B*: 1) were tested for growth on the five selective media. Isolate characterization included identification by MALDI-TOF MS (Bruker Daltonic GmbH, Bremen, Germany), susceptibility testing by Vitek2 (bioMérieux) and molecular characterization by the Anyplex VanR-Real-Time Detection Kit (Seegen, Düsseldorf, Germany).

In total, 4 out of 5 selective media (chromID VRE, CHROMagar, BrillianceVRE, VRESelect) detected all 105/105 VREfm after 24 h (chromID VRE, CHROMagar, BrillianceVRE) and 48 h (VRESelect), respectively. The fifth, ChromaticVRE, detected 104/105 strains. None of the 5 media detected all VVE-B strains, with strains detected after 24 h of incubation ranging between 7/14 (ChromaticVRE) and 8/14 (chromID VRE, CHROMagar, BrillianceVRE, VRESelect). Numbers remained the same after 48 h of incubation. Overall, 48-h incubation mainly increased growth of VSE on each agar, except chromID VRE, which did not increase unspecific growth (Table 1). The 10 *E. coli* isolates used as additional negative controls grew on neither of the 5 tested media. The within-species positive control (DSMZ 17,050 strain: VREfm) grew on all selective media after 24 h and 48 h of incubation. The negative control (DMSZ 20,477 strain: VSE) grew on neither of the agar.

The growth score (GS) was calculated as the sum of the growth in each smear (first, second and third smear) for each of the subgroups of the strains. This allowed for comparison of growth effectiveness between the different selective media and *E. faecium* subgroups. After 24 h of incubation, it was highest for all vancomycin-resistant enterococci (VREfm + VVE-B) in CHROMagar VRE (329) and lowest in Chromatic VRE Liolichem (228). It increased for all media on the second incubation day (Table 1).

Highest overall sensitivity was achieved by chromID VRE, CHROMagar VRE and Brilliance VRE (each 95.0%) at 24 h and remained constant at 48 h. VRESelect reached 95.0% sensitivity only at 48 h, while Chromatic VRE demonstrated the lowest sensitivity of all selective agar even after 48 h of incubation (93.3%, Table 2). Specific sensitivity for VVE-B was low in all agar and did not differ between 24 and 48 h. It was lowest in Chromatic VRE (50.0%, 7/14) and 57.1% (8/14) in all the others (Table 2). Of the 7 strains that evaded detection of at least one agar, 6 of those strains grew on neither of the tested selective media.

Highest overall specificity was observed by Chromatic VRE at 24 h (100%) but decreased slightly at 48 h (98.5%). Only the chromID VRE maintained a constant high specificity at 24 and 48 h (98.5%), while all other agar decreased in specificity at 48 h (Table 2).

To evaluate the balance between sensitivity and specificity, next, the Youden index (YI = sensitivity + specificity) − 1) was calculated. It was highest for chromID VRE and CHROMagar (each 0.94) at 24 h but only chromID maintained it at 48 h. It was lowest for Brilliance VRE at 48 h (0.89) (Table 2).

The highest negative predictive value was 0.92 at 24 h by chromID VRE, CHROMagar VRE, Brilliance VRE and at 48 h by VRE Select. It was lowest in Chromatic VRE at 24 h. The positive predictive value ranged from 1.00 (Chromatic VRE) to 0.97 (Brilliance VRE) after 24 h and between 0.99 (Chromatic VRE, chromID VRE) and 0.97 (BrillianceVRE, VRESelect) after 48 h of incubation (Table 2).

In order to assess the minimum number of bacteria within the sample needed for detection via each of the selective media, the limit of detection (LoD) was determined by a dilution series in pure cultures, as well as spiked human faecal mixtures. The required number of colony forming units (CFU)/mL was then calculated. Lowest LoD overall (pure culture, stool suspension, both 24 and 48 h) was determined for the CHROMagar and VRESelect which produced stable results (1.5 × 10^1^ CFU/mL) (Table 3). LoD for Chromatic VRE was only definable for pure cultures (24 h: 1.5 × 10^4^, 48 h: 1.5 × 10^2^). Inoculation with the stool suspension did not produce evaluable results even after four repetitions as unspecific growth of green colour covered the entirety of the agar (Supplementary Figures S1–S3): Since the manufacturer describes VREfm growing in blue-green colours, colonies were subcultivated and subsequently identified as different species, including Enterobacterales, via MALDI-TOF.

Since rectal swabs are more feasible for screening in clinical daily routine, the experiments were the repeated with the spiked human faecal mixture and rectal swabs. Lowest LoD after 24-h incubation was achieved by 2/5 agar plates (CHROMagar VRE, Chromatic VRE) with the minimum CFU within the sample being 1.5 × 10^2^ CFU/mL. For the remaining three agar plates (Brilliance VRE, VRESelect, chromID VRE) the LoD was 1.5 × 10^3^ CFU/mL. In total, 3 cultures growing at a dilution of 1:10^−7^ on Chromatic VRE after 48 h were identified as Ligilactobacillus salivarius by MALDI-TOF. LoD improved in 2/5 agar (VRESelect, chromID VRE) after 48-h incubation. It was the same in the three remaining selective media.

Overall, LoDs determined by our artificial rectal swabs were at least one dilution stage higher after 24-h incubation in comparison to the LoDs of our stool suspensions and pure culture suspensions (Table 3).

## 3. Discussion

In this study we compared five of the VRE screening media commercially available in Germany regarding their sensitivity and specificity. While overall performance was good for all media according to the manufacturers’ information, none of them were able to detect all *E. faecium* strains harbouring a *vanB* gene. This concurs with previous findings [39], although overall detection rate was lower in our study. This may be attributed to the relevant number of VVE-B tested in our study.

All agar plates displayed acceptable performance when looking at the VREfm isolates only, with sensitivity being high and in a similar range for all media. Reliability of results, as assessed by the Youden-Index, was also high for all agar plates. Furthermore, PPV was high in all agar. While these results cannot be directly ascribed to regions with low *vanB* VREfm, still a reliable detection can be expected.

However, only about 50%—7–8/14, respectively—of VVE-B strains were detected by the tested media. Since the detection limit of the selective media is derived from current breakpoints, such as a breakpoint 4 mg/L defined by the EUCAST, this finding must be expected. Yet, these results are a major issue for routine laboratory detection of VREfm. Even though a *vanA/B* PCR on every *E. faecium* isolate cultivated from clinical specimen would be optimal, it is not feasible for most diagnostic labs. Therefore, especially in patient screening, there will be a diagnostic gap of undetected vancomycin-resistant *E. faecium* strains.

Our findings indicate to not solely rely on VRE-selective media for detection of VVE-B, as several strains evaded detection via the five tested selective media. A combination of different selective media will most likely not enhance detection as six strains did not grow on either of the media. As these strains will evade detection irrespective of the agar used, a combination of methods should be considered, such as additional incubation in glycopeptide-containing broth or performing a *vanA/B* PCR on clinically relevant *E. faecium* isolates. Routine molecular exclusion of *vanA/B* seems advisable, if feasible, in clinical isolates of high importance that require immediate adequate antibiotic therapy strategies and in patients with antibiotic treatment failure.

While all selective media performed well, in regard to sensitivity and specificity with the chromID VRE, CHROMagar VRE and Brilliance VRE, a 24-h incubation period seems sufficient for detection of vancomycin-resistant *E. faecium,* especially since detection of VVE-B did not improve after 48 h of incubation. However, with the Chromatic VRE and VRESelect agar, a 48-h incubation may be justifiable.

Even though overall sensitivity increased only slightly in 2/5 agar plates, overall specificity decreased in 4/5 agars. On the other hand, the growth score increased for all selective media, thus potentially facilitating identification of VREfm on the second day. Yet, this comes with a higher risk of false positive results. As the use of same-day identification (e.g., via the MALDI-TOF technique) is widely implied in diagnostic laboratories, phenotypic species identification of *E. faecium* after an additional incubation period of 24 h would lead to a clinically relevant reporting delay.

Additionally, the LoD of the human faecal stool mixture, that is the number of CFU within the sample needed for detection, decreased only in one of the tested agar plates (ChromidVRE) after an additional 24 h of incubation (total of 48 h of incubation), while it remained stable for the remaining three agar. This further emphasizes that a 48-h incubation does not improve the detection level.

To mirror real-life screening settings in most hospitals we created artificial rectal swabs and determined LoD for each screening agar. Apart from the Chromatic VRE, all agar demonstrated a lower LoD after 24 and 48 h of incubation, implying screening stool specimens may be superior to rectal swabs to decrease detection gaps of VRE. As LoD from stool suspensions for Chromatic VRE was not determinable four times, comparison was only possible to pure cultures and the artificially spiked swabs.

We could not identify the reason for the unspecific growth when performing the LoD testing in stool mixture on Chromatic VRE. Despite repeating the experiment four times we were not able to obtain evaluable results. Yet, at the same time, none of the *E. coli* strains used as additional negative controls grew on the agar plates, indicating species-specificity for Enterococci. Despite some unspecific growth, experiments with the artificially spiked rectal swabs achieved evaluable results with the LoD being similar to all other tested media. Therefore, the unspecific growth can most likely be attributed to a batch-specific issue or improper storage during transport.

Next, these results also hint at an important aspect in regions with high *vanB* prevalence, such as Germany [3]. Although VVE carry the *vanA*-gene [22], there also seem to be *vanB* carrying strains with a vancomycin-susceptible phenotype [26], named VVE-B in this study. This must be considered, again especially in highly vital clinical specimen, when weighing potential diagnostic pathways and antibiotic therapies. Yet so far there is only limited data available on these strains.

For practical implementation, one limitation needs to be considered. As already mentioned, the examined samples did not comprise of clinical specimen, but rather of pure cultures, stool and swabs that were artificially inoculated with *E. faecium* strains. This was executed to ensure comparability and reproducibility of the results. It thus allowed for determination of further testing parameters, such as LoD.

In conclusion, while all agar showed a good performance, chromID VRE, CHROMagar VRE and Brilliance VRE had the highest sensitivity and specificity after 24 h of incubation. However, VVE-B may remain undetected even by molecular methods [15], yet a combination of different methods may decrease evasion rate. As stool samples demonstrated superiority to rectal swabs regarding LoD, we would recommend implementing stool samples as preferred screening specimen whenever accessible.

## 4. Materials and Methods

### 4.1. Selective Agar

All five selective agar commercially available in Germany at the time of the study were evaluated. They comprised of VRESelect™ (Bio-Rad, Hercules, CA, USA), CHROMagar™ VRE (CHROMagar, Paris, France), Brilliance™ VRE (Thermo Fisher, Dreieich, Germany), chromID^®^ VRE (bioMérieux, Marcy-l’Étoile, France) and Chromatic™ VRE (Liofilchem, Roseto degli Abruzzi, Italy).

### 4.2. Clinical Isolates

A total of 187 *E. faecium* strains from various clinical specimens (27 strains: blood culture, 24 samples: stool, 75 strains: urine, 70 strains: various clinical specimens, e.g., biopsies, punctates) were included in this study. Clinical specimens were incubated on non-selective media and stool samples on VRE-selective media (chromID^®^ VRE) for up to 48 h. Identification was performed by MALDI-TOF MS (Bruker Daltonic GmbH, Bremen, Germany) and vancomycin resistance was determined by Vitek2 (bioMérieux) in accordance with the current routine laboratory procedure. Vancomycin-resistance was defined in accordance with the EUCAST breakpoints (MIC > 4 mg/L).

**Figure 1:**
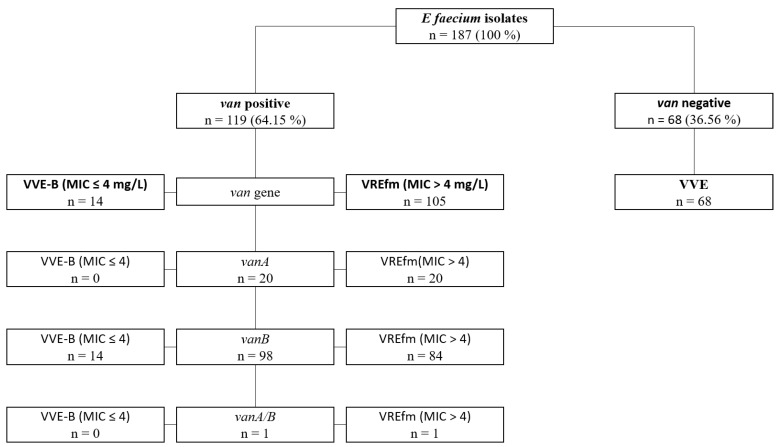
Overview of *E. faecium* strains. Overview of characteristics of all *E. faecium* strains included in the study. [VREfm: high-level vancomycin-resistant *E. faecium*; VVE-B: phenotypically tested vancomycin-susceptible *E. faecium* strains harbouring a *van*B-gene; VSE: vancomycin-susceptible *E. faecium*; MIC-minimal inhibitory concentration].

All isolates (Figure 1) were analysed for the presence of *vanA*/*vanB*/*vanC* by the Anyplex VanR Real-Time Detection Kit (Seegene, Düsseldorf, Germany). The limit of detection as given by the manufacturer is 2000 copies/reaction with no observable cross-reaction of the target between species. Isolates harbouring *vanA*/*vanB*/*vanC* with phenotypical susceptibility to vancomycin via Vitek2 were additionally investigated by broth microdilution using the MICRONAUT-S MRSA/GP plate (Merlin Diagnostika, Bornheim, Germany) confirming their vancomycin resistance.

Vancomycin-resistant *E. faecium* strains (*n* = 119) consisted of 20 *vanA,* 98 *vanB* and 1 *vanA*/*B* harbouring isolates. Of note, the latter strain harbouring a *vanA*/*B* gene was additionally confirmed by sequencing. The 68 (36.6%) vancomycin-susceptible strains tested negative for *vanA*/*vanB*/*vanC*105 strains expressed a vancomycin-resistance (84/105 *vanB*; 20/105 *vanA*; 1/105 *vanA*/*B*). The 14 remaining strains, while being vancomycin-susceptible, harboured a *vanB* gene, thus being defined as VVE-B (*vanB* positive, vancomycin-susceptible) in the following.

To assess specificity of the screening agar, 10 clinical *Escherichia* (*E.*) *coli* isolates—a gram-negative bacterium with high abundance in the human gut microbiota that should be suppressed by the selective media—were included in the testing. For within species control, two type strains were used: DMSZ 17,050 (VREfm) as positive control and DSMZ 20,477 (VSE) as negative control.

To obtain fresh cultures, all isolates were cultivated on SBA universal blood agar (Oxoid^TM^, Thermo Fisher Scientific; Waltham, MA, USA) 24 h prior to testing. Screening media were inoculated via the streak plate method with 10 µL of bacterial suspensions with a McFarland of 0.5 in NaCl (0.85%). They were then incubated at 37 °C ± 1 °C. Growth was assessed after 24 h and 48 h, respectively. Evaluation included the amount of growth (first, second or third smear), as well as the specific colour of the colonies in accordance with the manufacturer’s instructions. Since bacterial loads may vary substantially between and within clinical samples, such as rectal swabs, bacterial suspensions were used to ensure standardization and, thus, comparability.

The limit of detection (LoD) was determined in biological triplicates by three VREfm and two VVE-B strains. The LoD was defined as the highest dilution allowing for a minimum growth of three colonies on all three agar combined. A McFarland standard of 0.5 in NaCl was prepared from pure cultures (24 h) of each strain. Afterwards, serial dilutions from 1:10^−2^ to 1:10^−12^ were established. A total of 100 µL was then plated on each of the screening media with the *Eddy Jet* 2 W spiral plater (IUL Instruments S.A., Königswinter, Germany) and growth was evaluated after 24 and 48 h, respectively. Simultaneously, growth of each strain was recorded on Mueller-Hinton agar (Oxoid Deutschland GmbH, Wesel, Germany).

Furthermore, to test for specificity, LoD determination was additionally performed with a human faecal mixture sample: five VREfm-negative clinical stool samples were dissolved in NaCl (0.85%). They were then merged, in a ratio of 1:1, with each serial dilution of each strain. Prior to testing the stool samples, absence of VREfm was ensured via culture on chromID™ CPS^®^ Elite agar (bioMérieux, Marcy-l’Étoile, France), identification of Enterocci via MALDI-TOF, susceptibility testing of *E. faecium* isolates by Vitek2 and molecular testing for the presence of *vanA*/*vanB*/*vanC* by the Anyplex VanR Real-Time Detection Kit. LoD determination was performed as described above.

Subsequently, to assess typical screening conditions in hospitals, LoD determination from rectal swabs was performed. With the LoD of the human faecal mixture known, four dilution steps were chosen for testing the spiked rectal swabs (dilutions: 1:10^−4^–1:10^−7^) was repeated with rectal swabs. After preparation of the spiked faecal mixture samples as described above, swabs without medium (Sarstedt Group, Nürnsbrecht, Germany) were dipped into the mixture and then streaked out on each of the selective media. Growth was evaluated after 24 h and 48 h of incubation. Testing was carried out in biological triplicates with a new swab being used for each of the triplicates. LoD was then determined as described above.

### 4.3. Statistics

Molecular characterization was set as the gold standard for definition of vancomycin-resistance or susceptibility. The mean growth score (GS) was calculated for each isolate group (VREfm, VVE-B, VSE). For this, the number of smears showing growth were added up for each strain, thus varying between 0–3.

Overall sensitivity and specificity, positive (PPV) and negative (NPV) predictive values, and positive (LR+) and negative (LR−) likelihood ratios were calculated for each agar plate after 24- and 48-h incubation. Of note, as all strains harbouring a *van* gene were considered vancomycin-resistant, this group comprised of VVE-B and VREfm. Sensitivity for VVE-B and VREfm were also calculated separately for each group. In addition, to evaluate reliability, the Youden index (YI = sensitivity + specificity − 1) was calculated.

All values were calculated for *E. faecium* isolates only, thus excluding the 10 *E. coli* strains.

## Figures and Tables

**Table 1:** Overview of general growth of *E. faecium* on the agar plates *.

	Total no. of Strains of Each Category	ChromID VRE BioMérieux		CHROMagar VRE CHROMagar		Brilliance VRE ThermoFisher		VRESelect Bio-Rad		Chromatic VRE Liofilchem	
Incubation [Hours]		24	48	24	48	24	48	24	48	24	48
Growth											
No. of VREfm + VVE-B grown	119	113	113	113	113	113	113	112	113	109	111
No. of VREfm grown	105	105	105	105	105	105	105	104	105	102	104
No. of VVE-B grown	14	8	8	8	8	8	8	8	8	7	7
No. of VSE grown	68	1	1	1	3	3	4	2	3	0	1
No. of *E. coli* grown	10	0	0	0	0	0	0	0	0	0	0
Positive control (DSMZ 17050)	1	1	1	1	1	1	1	1	1	1	1
Negative control (DSMZ 20477)	1	0	0	0	0	0	0	0	0	0	0
Growth score (GS)											
GS VREfm + VVE-B	119	315	336	328	334	329	333	288	324	228	284
GS VREfm	105	297	312	304	310	306	310	268	300	215	268
GS VVE-B	14	18	24	24	24	23	23	20	24	13	16
GS VSE	68	1	1	1	3	5	6	2	3	0	1

* Growth score (GS) was calculated as the sum of all smears with growth. The *E. faecium* type strains DSMZ 17,050 (VREfm) and DSMZ 20,477 (VSE) were used as positive and negative control, respectively. [VREfm: vancomycin-resistant *E. faecium*; VVE-B: phenotypically tested vancomycin-susceptible *E. faecium* strains harbouring a *vanB*-gene; VSE: vancomycin-susceptible *E. faecium*].

**Table 2:** *E. faecium*-specific agar plate characteristics *.

	ChromID VRE (BioMérieux)		CHROMagar VRE (CHROMagar)		Brilliance VRE (ThermoFisher)		VRESelect (Bio-Rad)		Chromatic VRE (Liofilchem)	
Incubation [Hours]	24	48	24	48	24	48	24	48	24	48
Sensitivity [%] (95% CI)	95.0 (88.9–97.9)	95.0 (88.9–97.9)	95.0 (88.9–97.9)	95.0 (88.9–97.9)	95.0 (88.9–97.9)	95.0 (88.9–97.9)	94.1 (88.9–97.9)	95.0 (88.9–97.9)	91.6 (84.7–95.7)	93.3 (86.8–96.8)
Specificity [%] (95% CI)	98.5 (91.0–99.9)	98.5 (91.0–99.9)	98.5 (91.0–99.9)	95.6 (86.8–98.9)	95.6 (86.8–98.6)	94.1 (84.7–98.1)	97.1 (88.8–99.5)	95.6 (86.8–98.9)	100.0 (93.3–100.0)	98.5 (91.0–99.9)
Youden-Index	0.94	0.94	0.94	0.91	0.91	0.89	0.91	0.91	0.92	0.92
VREfm sensitivity [%]	100.0	100.0	100.0	100.0	100.0	100.0	99.1	100.0	97.1	99.1
VVE-B sensitivity [%]	57.1	57.1	57.1	57.1	57.1	57.1	57.1	57.1	50.0	50.0
PPV	0.99	0.99	0.99	0.97	0.97	0.97	0.98	0.97	1.00	0.99
NPV	0.92	0.92	0.92	0.92	0.92	0.91	0.90	0.92	0.87	0.89
LR+	64.57	64.57	64.57	21.52	21.52	16.14	32.00	21.52	n.c.	63.43
LR−	0.05	0.05	0.05	0.05	0.05	0.05	0.06	0.05	0.08	0.07

* Calculations include all tested 187 *E. faecium* strains excluding the two type strains used as control. [PPV: positive predictive value; NPV: negative predictive value; LR+: positive likelihood ratio; LR−: negative likelihood ratio; VREfm: high-level vancomycin-resistant *E. faecium*; VVE-B: phenotypically tested vancomycin-susceptible *E. faecium* strains harbouring a *vanB*-gene; n.c.: not calculatable].

**Table 3:** Limit of detection of the different selective agars *.

	Limit of Detection (CFU/mL)					
	24 h			48 h		
	Pure Culture	Stool Suspension	Artificial Rectal Swabs	Pure Culture	Stool Suspension	Artificial Rectal Swabs
CHROMID VRE (bioMérieux)	1.5 × 10^3^	1.5 × 10^2^	1.5 × 10^3^	1.5 × 10^1^	1.5 × 10^1^	1.5 × 10^2^
CHROMagar VRE (CHROMagar)	1.5 × 10^1^	1.5 × 10^1^	1.5 × 10^2^	1.5 × 10^1^	1.5 × 10^1^	1.5 × 10^2^
Brilliance VRE (ThermoFisher)	1.5 × 10^1^	1.5 × 10^1^	1.5 × 10^3^	1.5 × 10^1^	1.5 × 10^1^	1.5 × 10^3^
VRESelect (Bio-Rad)	1.5 × 10^3^	1.5 × 10^2^	1.5 × 10^3^	1.5 × 10^1^	1.5 × 10^2^	1.5 × 10^2^
Chromatic™ VRE (Liofilchem)	1.5 × 10^4^	-	1.5 × 10^2^	1.5 × 10^2^	-	1.5 × 10^2^

* For the limit of detection, the minimum number of bacteria needed within the sample to be detected was calculated as CFU/mL. [CFU: colony forming units].

## Data Availability

The data presented in this study are available on request from the corresponding author.

## Supplementary Materials

The following supporting information can be downloaded at: https://www.mdpi.com/article/10.3390/antibiotics12040666/s1, Figure S1: Exemplary presentation of serial dilution in biological triplicates for determination of limit of detection (LoD) (frontside of agar plates); Figure S2: Exemplary presentation of serial dilution in biological triplicates for determination of limit of detection (LoD) (backside of agar plates); Figure S3: Exemplary presentation of serial dilution of isolate 3 in a stool suspension for determination of limit of detection (LoD) on Chromatic™ VRE (Liofilchem^®^) (frontside of agar plates).
