# Supplementary material for: Comparison of Five Different Selective Agar for the Detection of Vancomycin-Resistant Enterococcus faecium
Source: Antibiotics (Basel). 2023 Mar 29;12(4):666. doi: 10.3390/antibiotics12040666 (PMC10135216; doi:10.3390/antibiotics12040666)

Comparison of Five Different Selective Agar for the Detection of Vancomycin-Resistant *Enterococcus faecium*

Alessa L. Boschert [1], Franca Arndt [1,2], Axel Hamprecht [1,3,4], Martina Wolke [1] and Sarah V. Walker [1,3,5,*]

Supplementary Data

Supplementary Figure S1:
Exemplary presentation of serial dilution in biological triplicates for determination of limit of detection (LoD) (frontside of agar plates); a-b: isolate 3, stool suspension 1.5x10^{-8} on Mueller-Hinton agar; d-f: : isolate 3, pure culture 1.5x10^{-8} on Mueller-Hinton agar; g-i: : isolate 3, stool suspension 1.5x10^{-8} on Chromatic™ VRE (Liofilchem®)

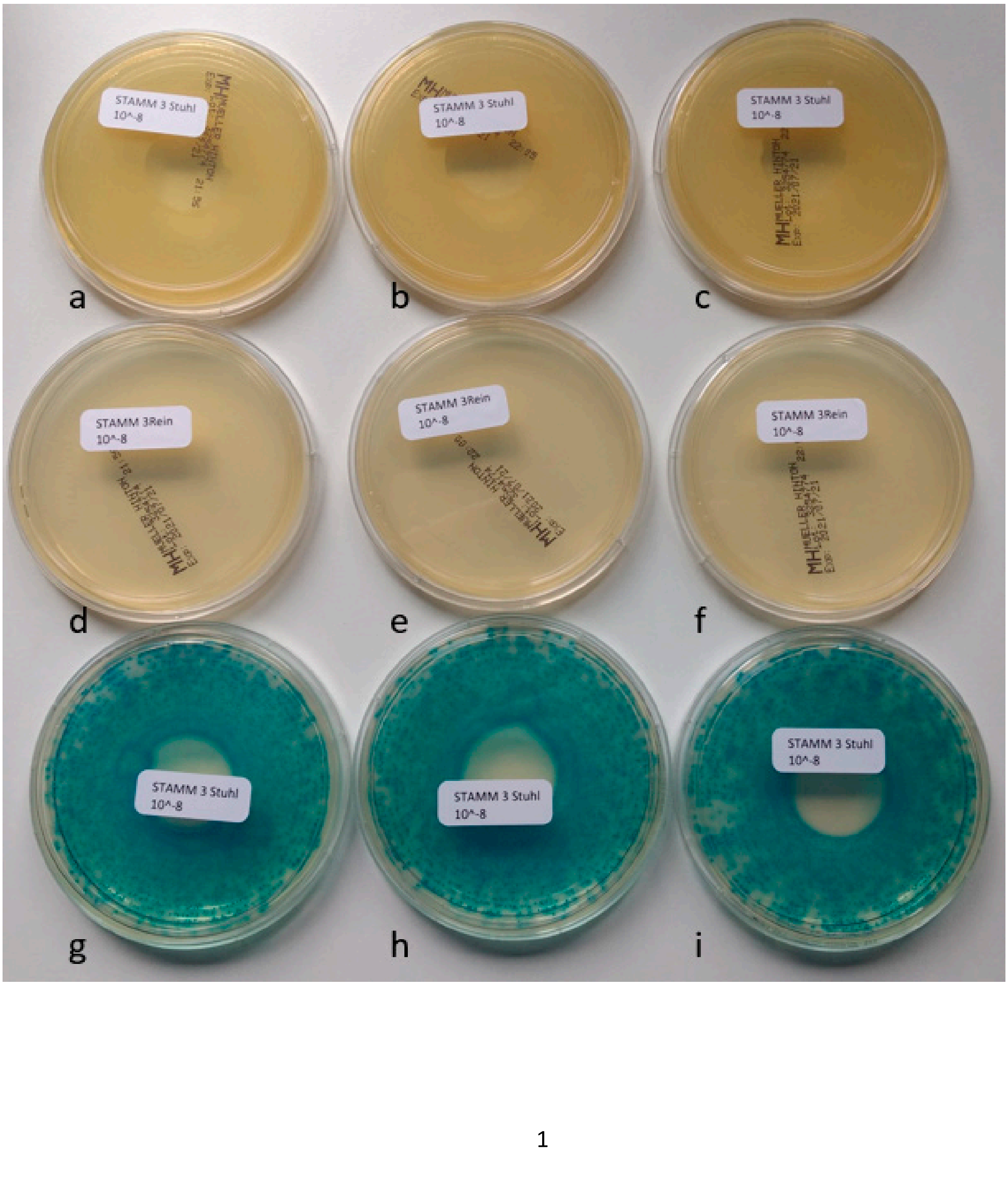

Supplementary Figure S2:
Exemplary presentation of serial dilution in biological triplicates for determination of limit of detection (LoD) (backside of agar plates); a-b: isolate 3, stool suspension $1.5x10^{-8}$ on Mueller-Hinton agar; d-f: : isolate 3, pure culture $1.5x10^{-8}$ on Mueller-Hinton agar; g-i: : isolate 3, stool suspension $1.5x10^{-8}$ on Chromatic™ VRE (Liofilchem®)

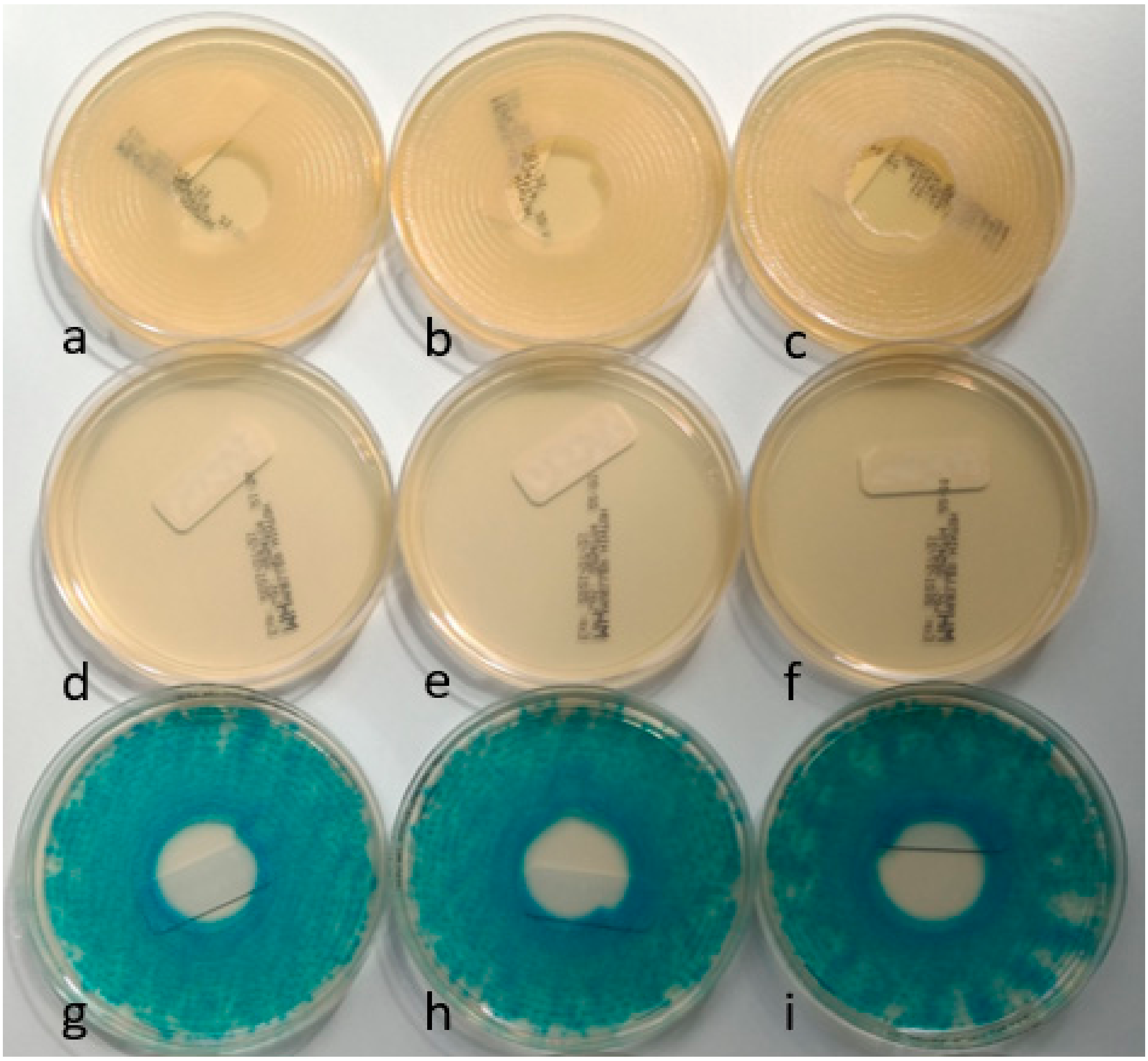

Supplementary Figure S3:

Exemplary presentation of serial dilution of isolate 3 in a stool suspension for determination of limit of detection (LoD) on Chromatic™ VRE (Liofilchem®) (frontside of agar plates); a: 1.5x10^{-2}; b: 1.5x10^{-4} ; c: 1.5x10^{-6}; d: 1.5x10^{-8}; e: 1.5x10^{-10}; f: 1.5x10^{-12}

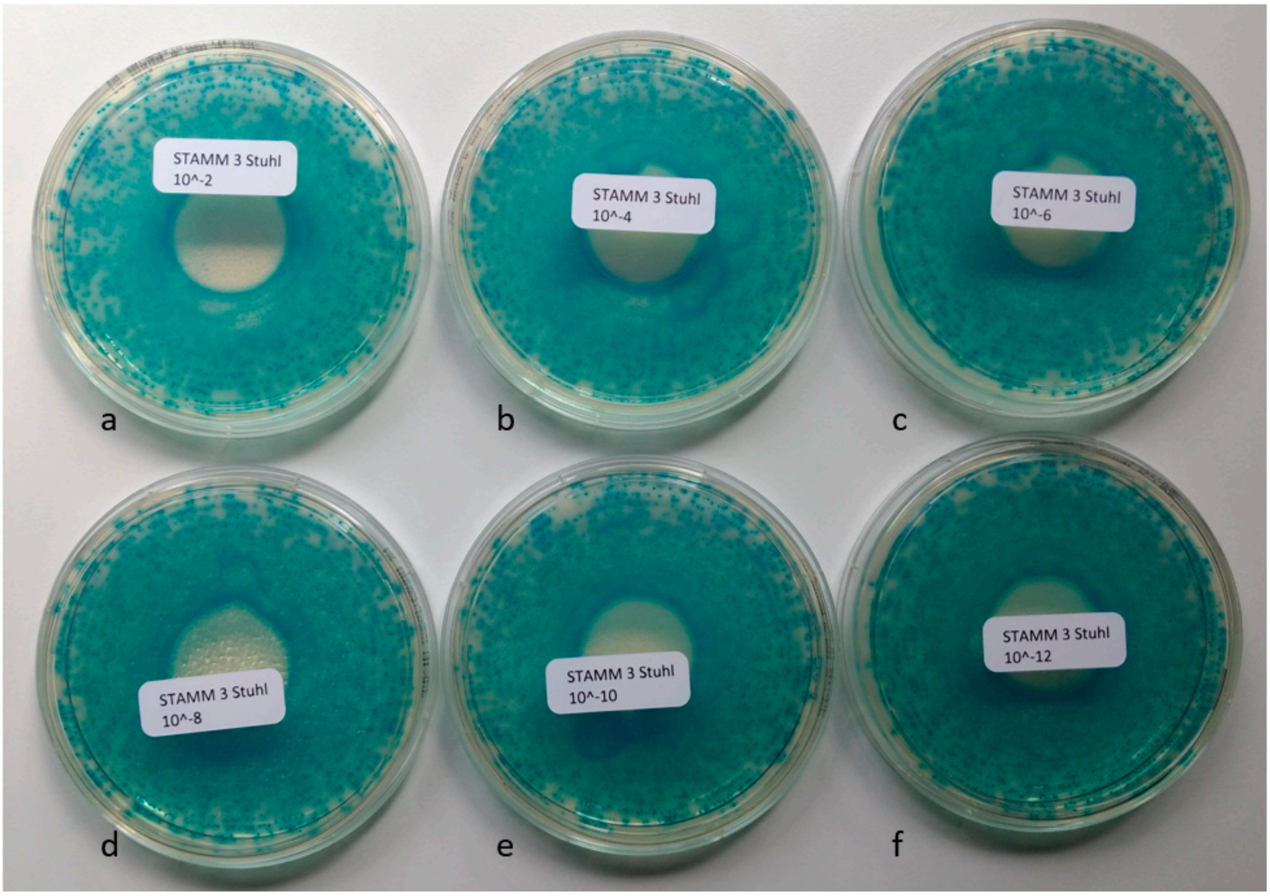

Supplement: Supplementary file 1 [file antibiotics-12-00666-s001.zip › antibiotics-2293099-supplementary.pdf]
